# PIM1 is responsible for IL-6-induced breast cancer cell EMT and stemness via c-myc activation

**DOI:** 10.1007/s12282-019-00966-3

**Published:** 2019-04-15

**Authors:** Xueqiang Gao, Xiangping Liu, Yangyong Lu, Yu Wang, Weihong Cao, Xiaoyi Liu, Haiyan Hu, Haibo Wang

**Affiliations:** 1 Breast Disease Center, The Affiliated Hospital of Qingdao University, 59 Haier Road, Qingdao, 266000 Shandong China; 2 Medical Research Center, The Affiliated Hospital of Qingdao University, Qingdao, Shandong 266000 China; 3 Department of Galactophore Surgery, Qingdao Women and Children’s Hospital, Qingdao, Shandong 266000 China

**Keywords:** PIM1, c-Myc, IL-6, EMT, Breast cancer

## Abstract

**Background:**

Interleukin-6 (IL-6) has been demonstrated to be a critical factor for breast cancer malignancy. However, the molecular mechanisms by which IL-6 induce breast cancer cells epithelial–mesenchymal-transition (EMT) and stemness remain elusive.

**Methods:**

Breast cancer cell lines T47D and MCF7 were exposed to IL-6, the expression of PIM1 was examined by quantitative real-time PCR (qRT-PCR) and western blot. Luciferase reporter assay was used to determine the transcriptional modulation of PIM1 by IL-6 and STAT3 inhibitor. Transwell assay was used to detect the invading ability of breast cancer cells induced by IL-6 or PIM1. The expressions of EMT and stemness markers were determined by qRT-PCR.

**Results:**

IL-6 promoted PIM1 expression in a dose- and time-dependent manner, and this induction could be abrogated by inhibiting STAT3 activation, subsequently suppressing the transcriptional level of PIM1. Moreover, we noticed that knocking down of PIM1 in cells which was exposed to IL-6 significantly impaired the invasion ability and the expression of EMT and stemness markers. On the contrary, overexpression of PIM1 promoted cell invasion and upregulated the expression of EMT and stemness markers. In addition, we demonstrated that c-myc, the cofactor of PIM1, involved in the pro-oncogenic roles of PIM1. Knocking down of c-myc attenuated the PIM1-induced cell EMT and stemness.

**Conclusion:**

This study proposed the upregulation of PIM1 by IL-6 contributed to breast cancer cell aggressiveness and targeting PIM1 or c-myc could be novel approaches for breast cancer treatment.

## Introduction

Breast cancer is the most common cancer in women worldwide, and the second leading cause of cancer-related death, with the mortality of 626,679 annually [1]. Although various clinical achievements have been accomplished in the diagnosis and treatment of breast cancer, the overall survival of breast cancer in advanced stage remains poor [2, 3]. It has been demonstrated that breast cancer cells with high heterogeneity and metastasis were the major challenge for the improvement of prognosis [4, 5]. Thus, the better understanding of molecular mechanisms underlying the initiation and development of breast cancer is urgently needed.

Epithelial–mesenchymal-transition (EMT) is critical for cancer metastasis as well as the generation and maintenance of cancer stem cells, contributing to cell self-renewal and resistance to therapy [6, 7]. The inflammatory microenvironment around the tumor plays pivotal role on EMT and stemness, and is characterized as a hallmark of cancer [8]. For example, IL-1β was reported to promote stem cell and EMT phenotypes by mediating ZEB1 in colorectal cancer cells and TGF-β promoted CCL22 expression and metastasis of hepatocellular carcinoma by miR-34a [9, 10]. Among these cytokines, IL-6 is an important mediator which can be secreted by immune, fibroblasts and cancer cells [11, 12]. IL-6 levels are increased in serval types of cancers including breast cancer, liver cancer and lung cancer [13]. Previous studies have reported that IL-6 induced CD44^+^ cells with stem-like and EMT properties in breast cancer [14, 15]. However, the contributions and mechanisms of IL-6 on the EMT and stemness of breast cancer have not been fully elucidated.

Proto-oncogene PIM1 belongs to the serine/threonine kinase family, which can phosphorylate a variety of targets, therefore, regulating cellular processes such as cell cycle, apoptosis, metabolism and inflammatory response. PIM1 has been demonstrated to be overexpressed and a potential biomarker in serval types of cancers, such as pancreatic cancer, colorectal cancer and acute myeloid leukemia [16–18]. PIM1 is thought to promote the carcinogenesis by cooperating with myc. PIM1 inhibitor upregulated p27 expression and nuclear accumulation, consequently suppressing tumor formation [19]. However, it is still elusive about the role of PIM1 in breast cancer. Previous report indicated that PIM1 was regulated by estrogen signaling and contributed to the growth of breast cancer cells [20]. Meanwhile, in triple-negative breast cancer (TNBC), PIM1 inhibition impaired the growth of both cell line and patient-derived xenografts and sensitized tumor cells to chemotherapy, partially via regulating myc and downstream Mcl1 [21]. Clinically, elevated PIM1 was associated with high tumor grade. Hence, PIM1 acted as a potential target for breast cancer therapy.

In the present study, we indicated that PIM1 was transcriptional activated by IL-6/STAT3 axis and was critical for IL-6 induced breast cancer cell EMT and stemness. Furthermore, we showed that PIM1 could promote cell EMT and stemness, which was related to the c-myc expression. Taken together, our data suggested that PIM1 might be a target for IL-6 induced breast cancer cell EMT and stemness.

## Materials and methods

### Cell lines and reagents

Human breast cancer cell lines T47D and MCF7 were purchased from the American Type Culture Collection (ATCC, Rockville, MD, USA). Cells were cultured in Dulbecco’s Modified Eagle’s Medium (DMEM, Gibco, Grand Island, NY, USA) supplemented with 10% Fetal Bovine Serum (FBS, Gibco) and penicillin/streptomycin. Cells were maintained in humidified incubator at 37 °C with 5% CO_2_. IL-6 (HY-P7044), STAT3 inhibitor WP1066 (HY-15312) was obtained from MedChemExpress (Monmouth Junction, NJ, USA).

### Plasmid and small interfering RNAs (siRNAs) transfection

The pCDNA3.1 vector containing PIM1 cDNA was obtained from Hanbio (Shanghai, China) and the siRNAs targeting PIM1, c-myc were purchased from GenePharma (Shanghai, China). Plasmid or siRNAs were transfected into cells using Lipofectamine 2000 (Invitrogen, Carlsbad, CA, USA) according to the manufacturer’s recommendation. The sequences of siRNAs were as follows: for PIM1, si-1, 5′-GGAACAACAUUUACAACUC-3′, si-2, 5′-GAUAUGGUGUGUGGAGAUA-3′; for c-myc, si-1, 5′-CAUCAUCAUCCAGGACUGUAU-3′, si-2, 5′-CGAGCUAAAACGGAGCUUU-3′. Cells were further detected after 48 h transfection.

### Quantitative real-time PCR (qRT-PCR)

Total cell RNA was extracted by Trizol reagent (Invitrogen) according to the manufacturer’s recommendation. 200 ng RNA was used to synthesize the first-strand cDNA using PrimeScript RT kit (Takara, Dalian, China). Quantitative real-time PCR was performed using SYBR Premix Ex Taq (Takara) following the manufacturer’s protocol. The indicated gene expression was normalized to GAPDH by 2^−∆∆Ct^. The primers used were listed in Table 1.

**Table 1:** Primer sequences

Gene name	Forward (5′–3′)	Reverse (5′–3′)
PIM1	GAGAAGGACCGGATTTCCGAC	CAGTCCAGGAGCCTAATGACG
Snail	TCGGAAGCCTAACTACAGCGA	AGATGAGCATTGGCAGCGAG
N-cadherin	TCAGGCGTCTGTAGAGGCTT	ATGCACATCCTTCGATAAGACTG
Twist	GTCCGCAGTCTTACGAGGAG	GCTTGAGGGTCTGAATCTTGCT
Oct4	GGGAGATTGATAACTGGTGTGTT	GTGTATATCCCAGGGTGATCCTC
Sox2	TACAGCATGTCCTACTCGCAG	GAGGAAGAGGTAACCACAGGG
Aldh1a1	CTGCTGGCGACAATGGAGT	CGCAATGTTTTGATGCAGCCT
GAPDH	CTGGGCTACACTGAGCACC	AAGTGGTCGTTGAGGGCAATG

### Western blot

Cells were lysed by RIPA buffer containing protease and phosphatase inhibitor cocktail (Roche, Mannheim, Germany) as described previously [22]. Total protein was measured by BCA methods. 50 µg protein was loaded on and separated by 10% SDS-PAGE and then transferred to PVDF membranes. The membranes were incubated with 5% bovine serum albumin (BSA) and primary antibodies overnight at 4 °C. After washing and incubating secondary antibody, the bands were visualized by ECL methods. The primary antibodies used were as follows: PIM1 (ab54503), GAPDH (ab8245), E-cadherin (ab1416), vimentin (ab8978) came from Abcam (Cambridge, MA, USA); c-myc (5605), p-STAT3 (94994), STAT3 (9139) came from Cellsignal Technology (Beverly, MA, USA).

### Luciferase reporter assay

The promoter region (− 1231 bp to + 155 bp from TSS) of PIM1 was cloned into pGL3 basic vector (Promega, Madison, WI, USA). The luciferase reporter assay was performed by co-transfecting the pGL-3-PIM1 and pRL-TK into T47D and MCF7 cells. After 24 h transfection, cells were treated with 10 µM WP1066 for 6 h in the presence and the absence of 10 ng/ml IL-6. The luciferase activities were measured using Dual-Luciferase Reporter Assay system (Promega) and luminometer.

### Transwell assay

The cell invasion was detected by Transwell chamber precoated with Matrigel (BD Bioscience, San Jose, CA, USA). Cells were seeded into the top chamber in serum-free medium and the low chamber contained medium with 10% FBS. After 48 h culturing, invaded cells were stained with crystal violet and photographed under phase contrast microscope. Cell numbers were measured by randomly selecting five fields.

### Statistical analysis

Statistical analysis was performed using GraphPad Prism 6 software. All experiments were performed independently at least three times. Data were presented as mean ± SEM. Student’s *t* test was used to determine the difference between each group. *P* value less than 0.05 was considered statistically significant.

## Results

### PIM1 is upregulated by IL-6 in breast cancer cells

**Fig. 1:**
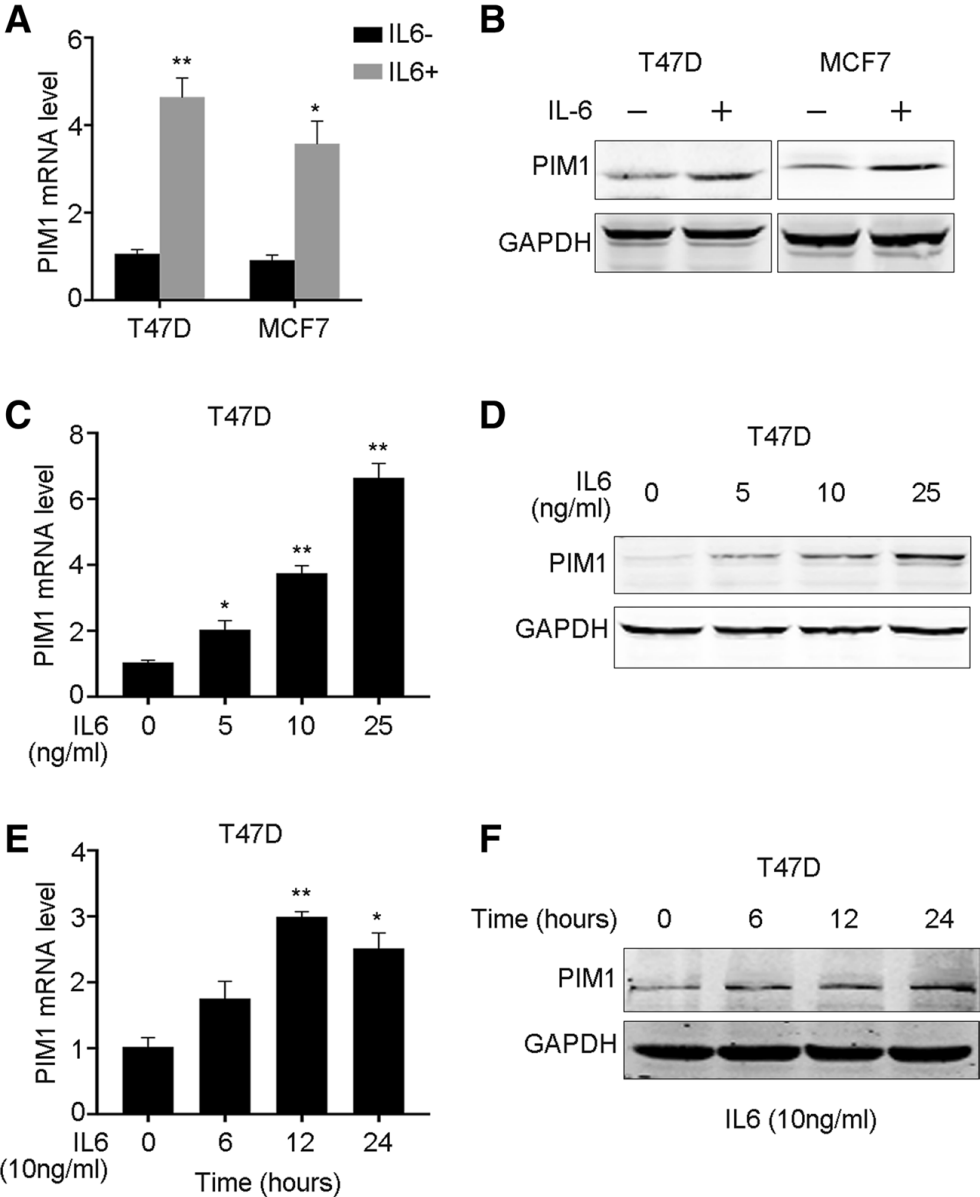
IL-6 induces PIM1 expression in breast cancer cells. **a** T47D and MCF7 cells were exposed to 10 ng/ml IL-6 for 24 h. mRNA level of PIM1 was examined by qRT-PCR. **b** The protein level of PIM1 was examined by western bolt after IL-6 treatment. The mRNA (**c**) and protein (**d**) levels of PIM1 in T47D cells treated with IL-6 in gradient concentration were examined by qRT-PCR and western blot, respectively. The mRNA (**e**) and protein (**f**) levels of PIM1 in T47D cells treated with 10 ng/ml IL-6 for indicated time were examined by qRT-PCR and western blot, respectively

We determined whether IL-6 induced PIM1 in breast cancer cell lines, leading to the increased expression of PIM1. PIM1 mRNA level was explored using quantitative PCR and we found that IL-6 could significantly elevate the mRNA of PIM1 in T47D and MCF7 breast cancer cells (Fig. 1a). Meanwhile, western blot assay demonstrated that PIM1 was enhanced by IL-6 in protein level as well (Fig. 1b). We also determined IL-6 induced PIM1 expression in a dose- and time-dependent manner (Fig. 1c–f). The increased mRNA levels of PIM1 in dose-dependent were mirrored by the protein expression (Fig. 1c, d), while the mRNA of PIM1 came to a peak after 12-h treatment and the protein level was even during that time (Fig. 1e, f). Collectively, we noticed that IL-6 could enhance the expression of PIM1 in breast cancer cells.

### IL-6 upregulates PIM1 by activating STAT3 signaling

**Fig. 2:**
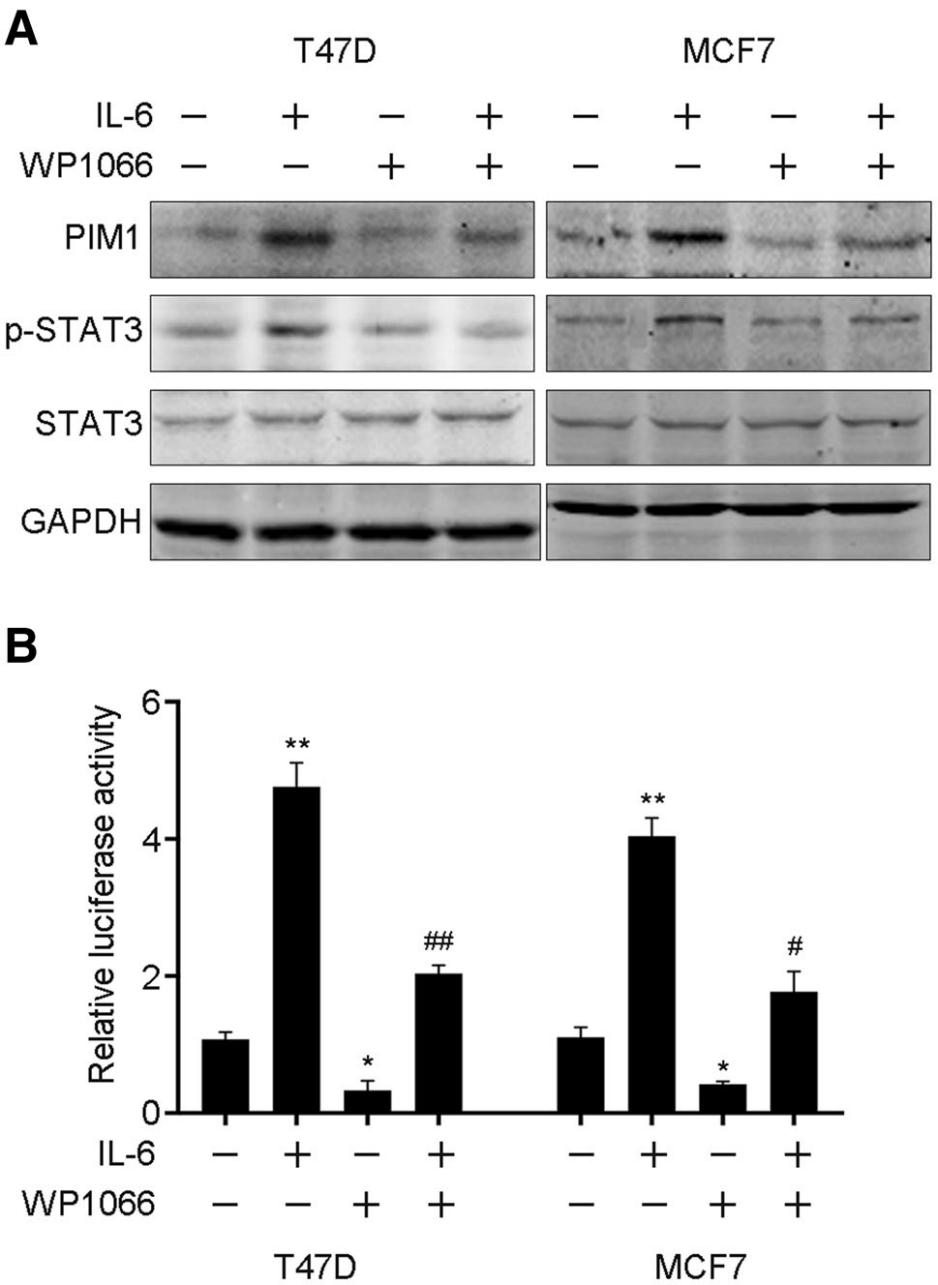
IL-6 transcriptional activates PIM1 by stimulating STAT3. **a** PIM1 and STAT3 status were examined by western blot after treating with WP1066 at the presence or absence of IL-6 stimulation in T47D and MCF7 cells. **b** Transcriptional activity of PIM1 in response to IL-6 was measured by luciferase reporter assay with or without STAT3 activation inhibition

As STAT3 is a vital transcription factor, which was primarily activated via IL-6, specific inhibitor WP1066 was used to identify the induction of PIM1 by IL-6. T47D and MCF7 cells were exposed to IL-6, and the expression of PIM1 was significantly upregulated. However, the upregulation of PIM1 was abrogated by treatment with WP1066, and the activation of STAT3 was decreased as well (Fig. 2a). Moreover, we cloned the promoter region of PIM1 and performed luciferase reporter assay in the presence of IL-6, or WP1066, or IL-6/WP1066. We noticed that PIM1 could be transcriptionally activated by IL-6 and further suppressed by WP1066 (Fig. 2b). Collectively, the results suggested that PIM1 was activated by IL-6/STAT3 signaling.

### Knocking down of PIM1 impairs IL-6-induced EMT and stemness

**Fig. 3:**
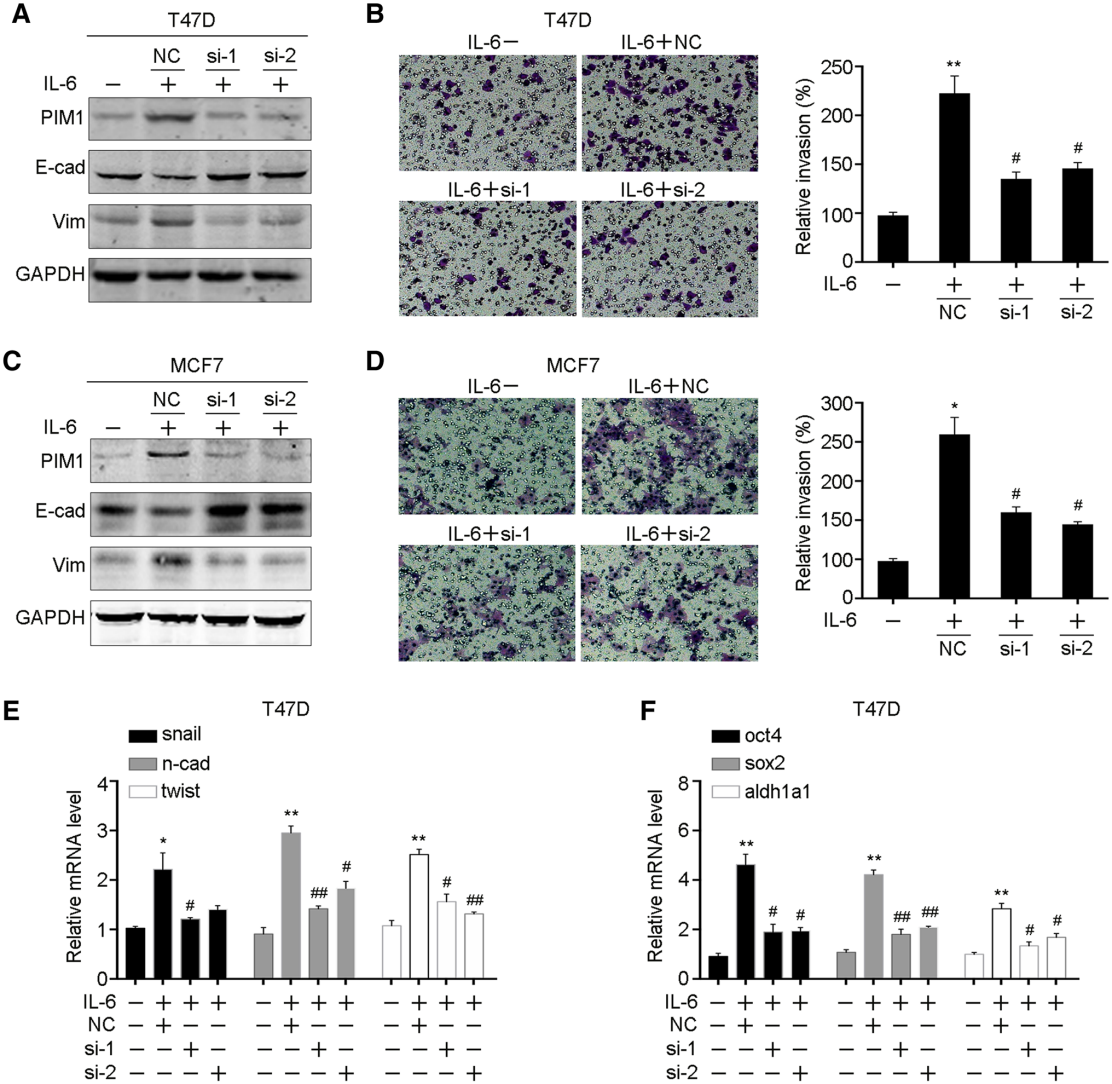
PIM1 is essential for IL-6-induced breast cancer cell EMT and stemness. **a** PIM1 and EMT markers were examined by western blot in IL-6-treated T47D cells followed by PIM1 knocking down. **b** Cell invasion ability was examined in IL-6-treated T47D cells followed by PIM1 knocking down. **c** Indicated proteins were examined in IL-6-treated MCF7 cells followed by PIM1 knocking down. **d** Cell invasion ability of IL-6-treated MCF7 cells followed by PIM1 knocking down. The mRNA levels of EMT markers (**e**) and stemness markers (**f**) were measured by qRT-PCR in IL-6-treated T47D cells followed by PIM1 knocking down. E-cad, E-cadherin; Vim, vimentin; n-cad, N-cadherin

As PIM1 was significantly upregulated by IL-6/STAT3 activation, we further investigated the roles of PIM1 in IL-6-induced breast cancer cell EMT and stemness. We performed siRNAs targeting PIM1 in T47D cells in the presence of IL-6 and found that PIM1 was significantly knocked down, together with increased E-cadherin and decreased Vimentin, which were both critical EMT markers (Fig. 3a). Meanwhile, the induction of invasion ability by exposing to IL-6 was also impaired by PIM1 knocking down (Fig. 3b). Consistently, similar results were found in the MCF7 cells, that knocking down of PIM1 attenuated the IL-6 induced cell EMT (Fig. 3c, d). Furthermore, we performed qRT-PCR to detect the expression of markers of EMT and stemness, including snail, N-cadherin, twist, oct4, sox2 and aldh1a1. We noticed that inhibition of PIM1 impaired the expression of these markers which were induced by IL-6 (Fig. 3e, f). Collectively, the results indicated that PIM1 was critical for IL-6-induced EMT and stemness.

### Overexpression of PIM1-induced EMT and stemness

**Fig. 4:**
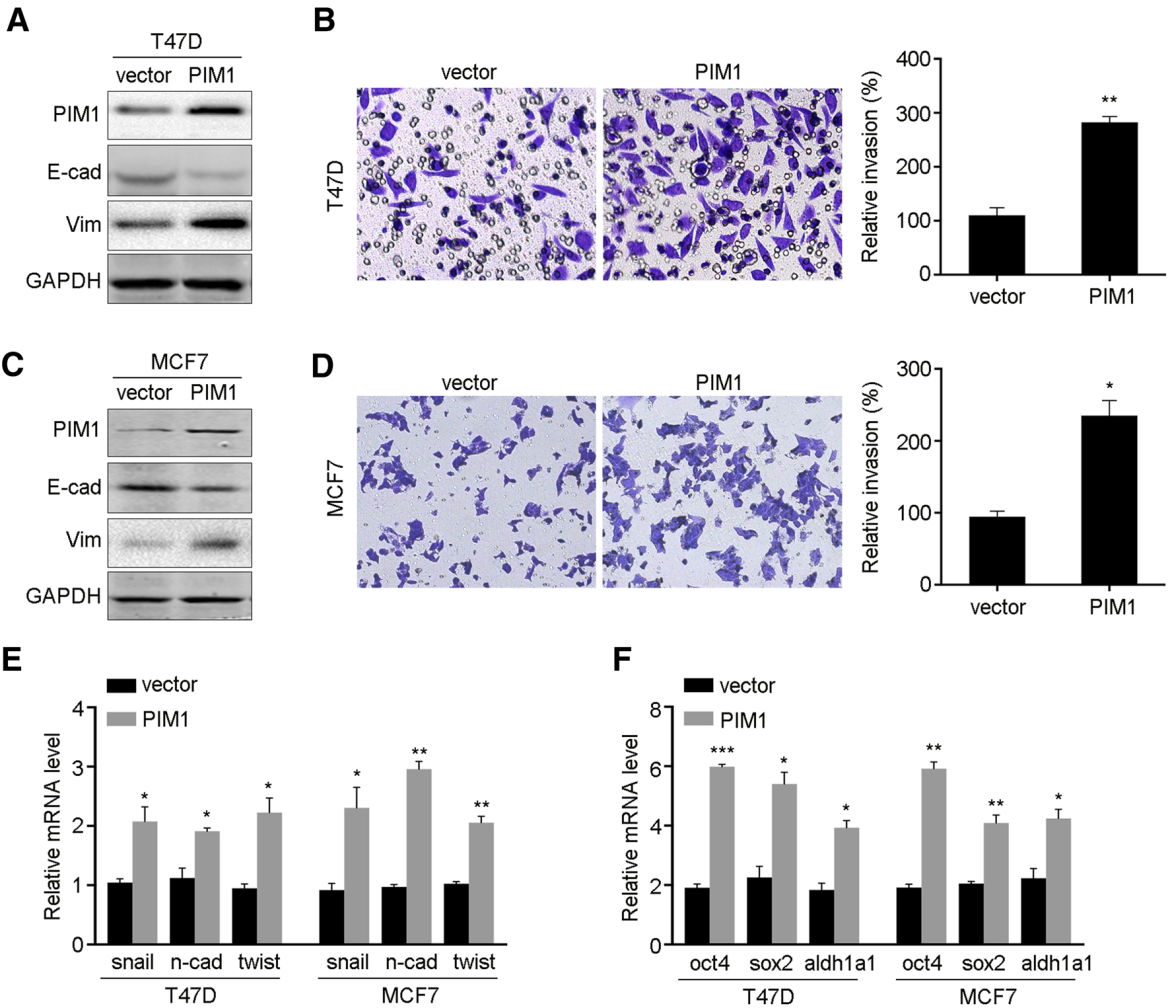
PIM1 facilitates cell EMT and stemness. **a** Overexpression of PIM1 was detected in T47D cells by western blot, together with EMT markers. **b** Cell invasion ability of T47D cells overexpressing PIM1. **c** Overexpression of PIM1 in MCF7 cells. **d** Cell invasion ability of MCF7 cells overexpressing PIM1. The mRNA levels of EMT markers (**e**) and stemness markers (**f**) were detected in T47D and MCF7 cells overexpressing PIM1

To further explore the function of PIM1 in cell EMT and stemness, ectopic expression of PIM1 was performed in T47D and MCF7 cells, the expression of E-cadherin was decreased while vimentin was increased (Fig. 4a, c). Also, PIM1 significantly facilitated the invading ability as demonstrated by Transwell assay (Fig. 4b, d). Then, we noticed that PIM1 increased the mRNA levels of the EMT and stemness markers (Fig. 4e, f). Thus, PIM1 is critical in promoting breast cancer cell EMT and stemness.

### C-myc is critical for PIM1-induced EMT and stemness

**Fig. 5:**
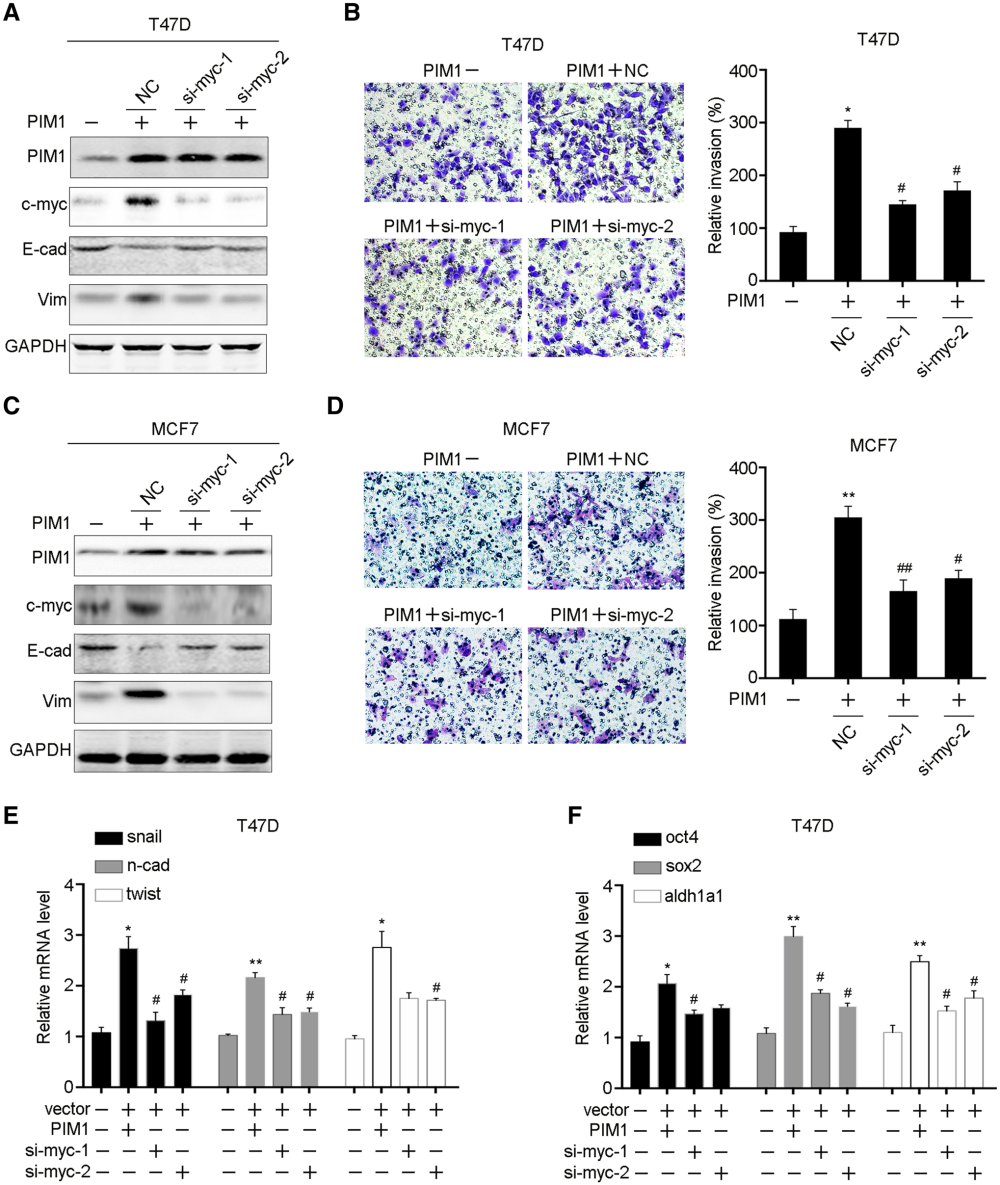
c-myc is involved in PIM1 promoting EMT and stemness. **a** Indicated proteins were examined by western blot in PIM1 overexpressing T47D cells followed by c-myc knocking down. **b** Cell invasion ability was examined in PIM1 overexpressing T47D cells followed by c-myc knocking down. **c** Indicated proteins were examined by western blot in MCF7 cells. **d** Cell invasion ability of PIM1-overexpressing MCF7 cells followed by c-myc knocking down. The mRNA levels of EMT markers (**e**) and stemness markers (**f**) were measured by qRT-PCR in PIM1-overexpressing T47D cells followed by c-myc knocking down. E-cad, E-cadherin; Vim, vimentin; n-cad, N-cadherin

Previous studies have implied that MYC was a target of PIM1 and PIM1 inhibition abrogated the growth of MYC-overexpressing tumors, we detected whether MYC was involved in PIM1-induced breast cancer EMT and stemness. We knocked down the expression of c-myc in T47D and MCF7 cells with PIM1 overexpression, respectively. C-myc and vimentin were significantly upregulated by PIM1 overexpression, and then decreased after c-myc knocking down (Fig. 5a, c). However, the expression of E-cadherin was restored after c-myc knocking down. Transwell assay also indicated that knocking down of c-myc attenuated PIM1-induced cell invading ability in T47D and MCF7 cells (Fig. 5b, d). Meanwhile, the makers of EMT and stemness were also decreased after c-myc knocking down (Fig. 5e, f). Taken together, these results indicated that c-myc was involved in PIM1-induced breast cancer cell EMT and stemness.

## Discussion

In the present study, our data identified that PIM1 was induced by IL-6 and participated in IL-6-mediated EMT and stemness in breast cancer. Functional analysis using ectopic overexpression and RNAi knockdown implied that PIM1 promoted breast cancer cell malignancy phenotypes.

Previous study indicated that PIM1 located on chromosome 6p21-p25, a recurrent amplicon, and the copy number amplification was responsible for the upregulation of PIM1 in breast cancer [23]. Here, we reported that IL-6 induced PIM1 expression in a time- and dose-dependent manner in T47D and MCF7 cells. Previous reported implied that IL-6 might link with the expression of PIM1 in pancreatic cancer cells and fibroblasts, and we observed this regulation pattern in breast cancer as well [24, 25]. Further exploration using STAT3 inhibitor demonstrated that IL-6 upregulated PIM1 via activation of STAT3 signaling, thereby, transcriptionally activating PIM1 expression. Emerging evidence has suggested that inflammatory microenvironment confers cancer cell EMT, metastasis and chemoresistance [26]. For example, myeloid-derived suppressor cells (MDSC) triggered miR-101 in cancer cells and subsequently repressed CtBP2, leading to the ovarian cancer cell stemness [27]. Also, TGF-beta converted CD44^−^ non-cancer stem cells (CSCs) into undifferentiated CD44^+^ CSCs in colorectal cancer, and IL-6 and IL-8 were important to maintain aggressive traits of breast cancer cells [28, 29]. Mechanically, previous reports indicated that IL-6 could regulated a bunch of key genes to promote cancer cell EMT and stemness, such as Fra-1 and NRF2 [30, 31]. Hence, our data for the first time, indicated that PIM1 was a downstream target of IL-6 and critical for breast cancer cell EMT and stemness.

There are some limitations of this study. We will employee more cell lines to determine the regulation of PIM1 both in hormone receptor-positive cells or TNBC cells. Also, further study will focus on illustrating the regulation and impact of PIM1 by IL-6 using in vivo mouse model. We could use PIM1 inhibitors or antibody against IL-6 to explore the anti-cancer effect.

PIM1, acted as an oncogene, has been demonstrated to regulate cell cycle, EMT and metastasis in many types of cancers including melanoma, hepatocellular carcinoma and osteosarcoma. PIM1 could interact and phosphorylate Smad2/3 and induce the expression of ZEB1, Snail and Twist [32]. Other well-known substrates of PIM1 included CDC25A, p21, c-TAK and FOXP3 [33, 34]. Meanwhile, miR-542, miR-486 and miR-124-3p were reported to target PIM1, as well as hypoxia and virus infection [35–37]. Recently, PIM1 was reported to induce the Warburg effect under glucose deprivation condition by upregulation of HK2 and LDHA and these results were linked with c-myc expression [33]. Horiuchi et al. [38] performed kinome-wide synthetic lethal shRNA screen in non-immortalized human mammary epithelial cells (HMECs) overexpressing c-myc to identify the druggable target for the c-myc, and PIM1 was found as the most significant target, as well as genes in NF-κB, ERK, PI3K/AKT and WNT signaling pathways. Knocking down of PIM1 significantly suppressed TNBC cell survival, while had little effect on the proliferation and death of hormone receptor-positive breast cell lines. However, we found that PIM1 induced breast cancer cell EMT and stemness, which might be independent of the hormone status. In addition, Wang et al. reported that PIM1 synergized with c-myc to induce prostate cancer progression [39]. Consistent with previous studies, c-myc was closely associated with PIM1 expression in our finding. Knocking down of c-myc in T47D and MCF7 overexpressing PIM1 cells significantly restored the expression of E-cadherin and suppressed vimentin expression, suggesting that c-myc was essential for PIM1-induced EMT and stemness.

In summary, our findings provided insight into the mechanism by which pro-inflammatory cytokine IL-6 induced breast cancer cell EMT and stemness and provided the potential approach to suppress the upregulation of PIM1 by IL-6. Moreover, we determined the biological functions of PIM1 in breast cancer, not only the growth but the metastasis and stemness, and supported the rationale that inhibition of PIM1 and c-myc could have clinical benefit for breast cancer patients.
